# MCSdb, a database of proteins residing in membrane contact sites

**DOI:** 10.1038/s41597-024-03104-7

**Published:** 2024-03-08

**Authors:** Xianrun Pan, Liping Ren, Yu Yang, Yi Xu, Lin Ning, Yibing Zhang, Huaichao Luo, Quan Zou, Yang Zhang

**Affiliations:** 1 College of Medical Technology, Chengdu University of Traditional Chinese Medicine, Chengdu, China; 2 School of Healthcare Technology, Chengdu Neusoft University, Chengdu, China; 3 School of Life Science and Technology, University of Electronic Science and Technology of China, Chengdu, China; 4 Glasgow College, University of Electronic Science and Technology of China, Chengdu, China; 5 Department of Clinical Laboratory, Sichuan Clinical Research Center for Cancer, Sichuan Cancer Hospital & Institute, Sichuan Cancer Center, Affiliated Cancer Hospital of University of Electronic Science and Technology of China, Chengdu, China; 6 Institute of Fundamental and Frontier Sciences, University of Electronic Science and Technology of China, Chengdu, China; 7 Innovative Institute of Chinese Medicine and Pharmacy, Academy for Interdiscipline, Chengdu University of Traditional Chinese Medicine, Chengdu, China

**Keywords:** Protein databases, Organelles

## Abstract

Organelles do not act as autonomous discrete units but rather as interconnected hubs that engage in extensive communication by forming close contacts called “membrane contact sites (MCSs)”. And many proteins have been identified as residing in MCS and playing important roles in maintaining and fulfilling specific functions within these microdomains. However, a comprehensive compilation of these MCS proteins is still lacking. Therefore, we developed MCSdb, a manually curated resource of MCS proteins and complexes from publications. MCSdb documents 7010 MCS protein entries and 263 complexes, involving 24 organelles and 44 MCSs across 11 species. Additionally, MCSdb orchestrates all data into different categories with multitudinous information for presenting MCS proteins. In summary, MCSdb provides a valuable resource for accelerating MCS functional interpretation and interorganelle communication deciphering.

## Background & Summary

**Fig. 1:**
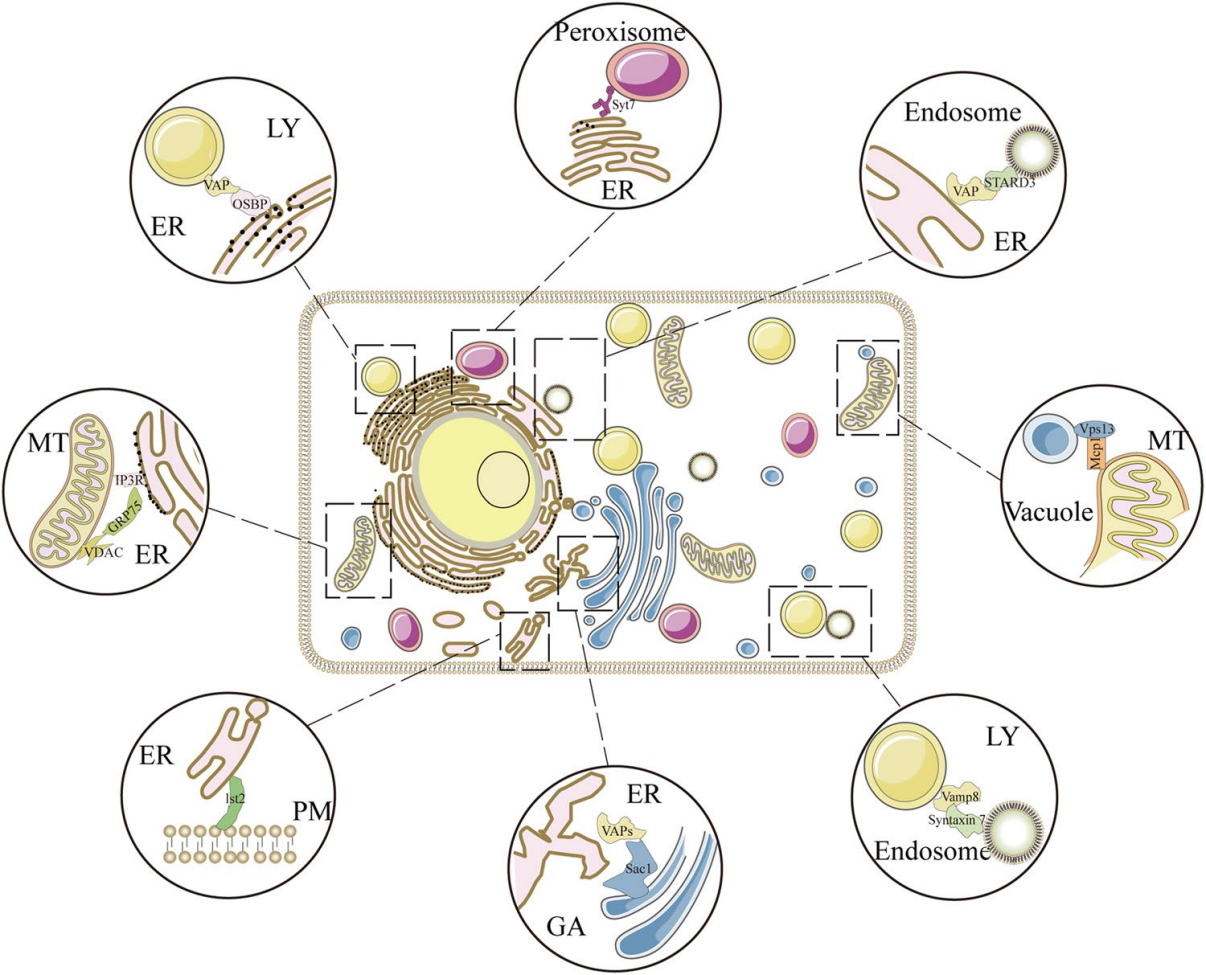
Schematic diagram of MCSs in eukaryotic cells.

“All things are mutually woven together and therefore have an affinity for each other”—Marcus Aurelius, *Meditations*. Most biologists would agree with Aurelius’ statement because connectivity is observed at every level of biology, occurring between biomolecules, cells, tissues and organisms^1–5^. Therefore, it is becoming increasingly evident that organelles do not act as autonomous discrete units but rather as interconnected hubs that engage in extensive communication by forming close contacts called “membrane contact sites (MCSs)”^6,7^. The MCS is defined as an area of close apposition (from 10 to 80 nm) between the membranes of two organelles that are physically connected via proteinaceous tethers but do not fuse (Fig. 1)^8,9^. Current studies on MCSs are moving toward the central stage in cell biology^9,10^. Multiple MCSs have been identified between virtually all organelles in eukaryotic cells and participate in various biological processes and intracellular signaling, such as autophagy, lipid metabolism, calcium homeostasis and organelle trafficking and remodeling^10–13^. Moreover, aberrant loss or gain of function of MCSs can contribute to various diseases, such as cancer, metabolic diseases and neurodegenerative disorders^14–18^. In a sense, studies on spatiotemporal coordination among organelles indicate the existence of a hidden world of cellular interorganelle communication networks connected by MCS waiting to be explored^19,20^.

As a key component of MCSs, the proteins residing in membrane contacts play a crucial role in maintaining and fulfilling functions specific to MCSs^9,21^. Understanding how these protein tethers and membrane contacts coordinate organelle function will redefine our view of the cell^14,22^. Recently, a growing number of MCS proteins have been identified and functionally characterized. For example, three Aster proteins (Aster-A, -B, -C) can be recruited to the plasma membrane (PM)-endoplasmic reticulum (ER) and facilitate nonvesicular plasma membrane to ER cholesterol transport^23^. The Sel1 L-Hrd1 protein complex is involved in ER-mitochondria (MT) crosstalk and can affect mitochondrial dynamics in brown adipocytes^24^. The protein complex consisting of SLPD1, SLPD2 and LIPA mediates lipid droplet (LD)-PM tethering in plant cells^25^. Loewen *et al*., identified a short conserved determinant called the FFAT motif. This motif interacts with the VAP protein family, which are conserved integral membrane proteins located in the ER. These proteins play a pivotal role in the formation and function of various ER-related MCSs^26–28^. Subsequently, a series of proteins that contain the FFAT motif were recognized as MCS proteins^29,30^. Several studies have begun to screen MCS proteins by combining traditional biochemical approaches (subcellular fractionation and pull-down) with mass spectrometry (MS)-based proteomics^31–33^. However, the limitations (e.g., destabilized contacts, contamination by other components) of such traditional biochemical approaches may lead to a large number of false-positive proteins being detected^9,34^. Nonetheless, some proximity labeling approaches combined with high-throughput proteomic analysis, such as BioID, Contact-ID, and Split-TurboID, have recently been developed for global mapping of MCS proteins and are promising for MCS proteomics studies^35–39^.

Although MCSs have received increasing attention and the proteins residing in MCSs have been extensively identified in the past few years^40–42^, an appropriative database for storing, integrating and reorganizing MCS proteins is still lacking. Therefore, we developed MCSdb, a manually curated database of experimentally supported MCS proteins and complexes from publications. The current version of MCSdb documents approximately 7000 manually curated MCS protein entries and 263 complexes with experimental evidence, involving 24 organelles and 44 MCSs across 11 species. Furthermore, MCSdb grades all MCS protein entries into 3 categories according to the confidence level of experimental evidence. MCSdb also provides multitudinous information to help query and analyze MCS proteins and complexes. To our knowledge, MCSdb is the first database specifically focusing on proteins located in MCS. We believe that this database will be invaluable in accelerating MCS functional research and interorganelle communication deciphering. Dataset of the MCS proteins and complexes is free available in Figshare^43^.

## Methods

### Data collection

**Fig. 2:**
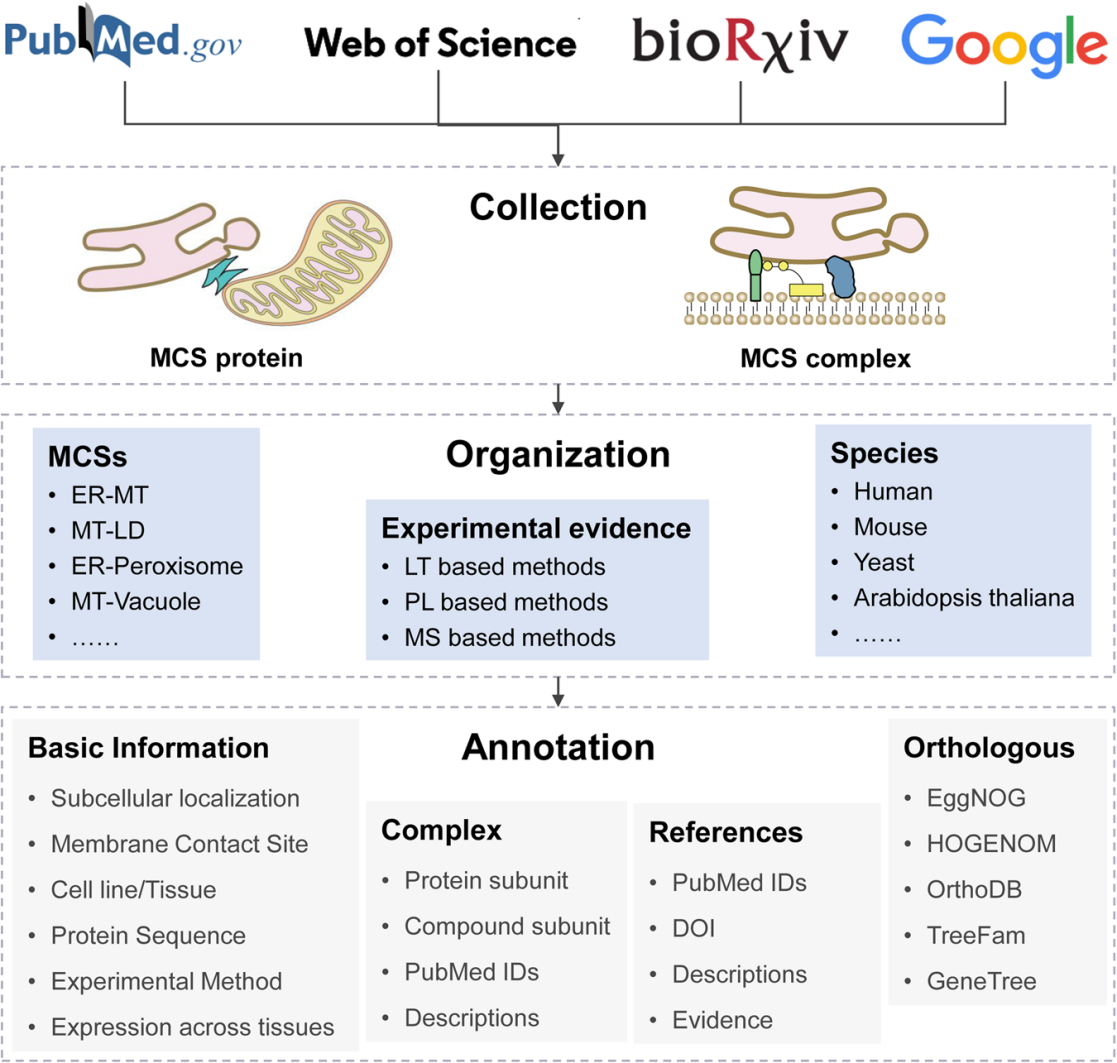
Data collection, organization and annotation of MCSdb.

The MCS proteins in the database were curated manually from the literature (before Jun. 2023). First, we retrieved literature from PubMed, bioRxiv, Web of Science and Google Scholar using the following keywords: ‘membrane contact site’, ‘organelle communication’, ‘organelle interaction’, ‘mitochondria-associated membranes’, ‘protein tether’, and ‘proximity labeling’. All binary phrases consist of two organelles: ‘endoplasmic reticulum-plasma membrane’, ‘endoplasmic reticulum-Golgi’, ‘endoplasmic reticulum–peroxisome’ and ‘endoplasmic reticulum-lipid droplet’ (Fig. 2). Then, all retrieved publications were preliminarily reviewed by expert curators to filter out false-positive papers. According to several review articles^9,10^, the MCS is defined as an area of close apposition (from 10 to 80 nm) between two bi- or mono-layer membrane-bound organelles that are physically connected via proteinaceous tethers but do not fuse. And to be included as an MCS protein in MCSdb, there must be experimental confirmation that the protein is located at the MCS, or evidence showing that it can be recruited to the MCS, contributing to its formation or to the functions associated with the MCS. Additionally, the protein complexes located and acting in MCSs are recorded in MCSdb.

### Data organization

First, we distinguished MCSs by the connected organelles of an MCS (named ER-PM, ER-MT and MT-LD, etc.), and a total of 44 MCSs were defined. Then, we divided all MCS protein entries into different categories according to the MCS of the proteins located (Fig. 2). Meanwhile, we graded all documented MCS protein entries into 3 categories: low-throughput (LT) experimental-based methods, proximity labeling (PL)-based methods and mass spectrometric (MS)-based methods. LT-based methods represent the proteins identified and functionally characterized by low-throughput experimental methods, and there are two additional inclusion criteria for LT-based proteins: (1) Proteins cannot be solely identified through high-throughput experiments; (2) The number of MCS proteins identified by the literature source for a given protein is less than 10 (inspiration from the protein-protein interaction (PPI) databases’ criteria: MINT^44^, mentha^45^, InWeb_InBioMap^46^). PL methods represent the proteins identified by combining PL approaches with high-throughput proteomics. MS methods represent the proteins identified by combining traditional biochemical approaches with MS techniques. To enhance user ability to evaluate the reliability of MS-based data, we introduce a scoring system anchored in protein subcellular localization and protein-protein interaction (PPI) networks. We sourced interaction information for MS-based proteins from the String database and subcellular localization data from the Uniprot database^47^ to ascertain if MS-based proteins and their interacting partners are situated within the MCS organelle. This system stratifies MS-based data into five confidence levels (L1 to L5), with detailed rules outlined on the “Help” page.

### Data annotation

To unify the proteins from multiple publications in authoritative reference databases, all MCS proteins were mapped to the NCBI gene database (Entrez ID)^48^ and UniProt (UniProt ID)^47^. Five compounds involved in MCS complexes were mapped to the PubChem database (PubChem CID)^49^. Information of subcellular localization, cell line/tissue and descriptions of MCS proteins was manually curated from the literature (Fig. 2). Human and mouse gene expression data across different tissues were collected from Human Protein Atlas (HPA) (62 human tissues)^50^ and the TISSUES 2.0 database (39 mouse tissues)^51^, respectively. Protein sequence data were collected from the UniProt database. Orthology information of MCS proteins was collected from five databases: EggNOG^52^, HOGENOM^53^, OrthoDB^54^, TreeFam^55^ and GeneTree^56^. The PPIs involved in MCS complexes were extracted from the bioGRID database^57^.

## Data Records

### Recorded datasets

MCSdb is free available at Figshare^43^. it provides four types of datasets. The first dataset consisted of detail information of all MCS proteins (xlsx file), including the Entrez ID, protein name, Synonyms, UniProt ID, species, MCS location, and the references (Experimental Method, Cell line/Tissue, PMID and Description and evidences). The second dataset consisted of detail information of all complexes (xlsx file), including complex name, subunit number, species, MCS location, and the information about all subunits (protein names and UniProt ID). And the detail information of the compound subunit was also provided (names, PubChem CID, Formula and SMILES). The third dataset consisted of list of 44 MCS locations and along with their corresponding organelles (xlsx file). The last dataset consisted of detail information of all literatures, including PMIDs, DOI, journal name, authors, title, abstract and published time.

The MCS proteins documented in the database were identified by various experimental methods and thus have different confidence levels. For example, some collected MCS proteins are high-confidence because they were well identified and functionally characterized by multiple low-throughput experimental methods, whereas some other proteins were only screened by the high-throughput method and require further experimental validation^9,34^. Therefore, after careful consideration of common perspectives from multiple review articles and the characteristics of the data^9,10,35–38,58,59^, we graded all documented MCS protein entries into 3 categories according to the confidence level of experimental evidence: LT experimental-based methods, PL-based methods and MS-based methods (confidence: LT-based methods > PL-based methods > MS-based methods). Please note that the data from MS-based methods have a high false positive rate. You are advised to use it with caution.

### Data statistics

**Fig. 3:**
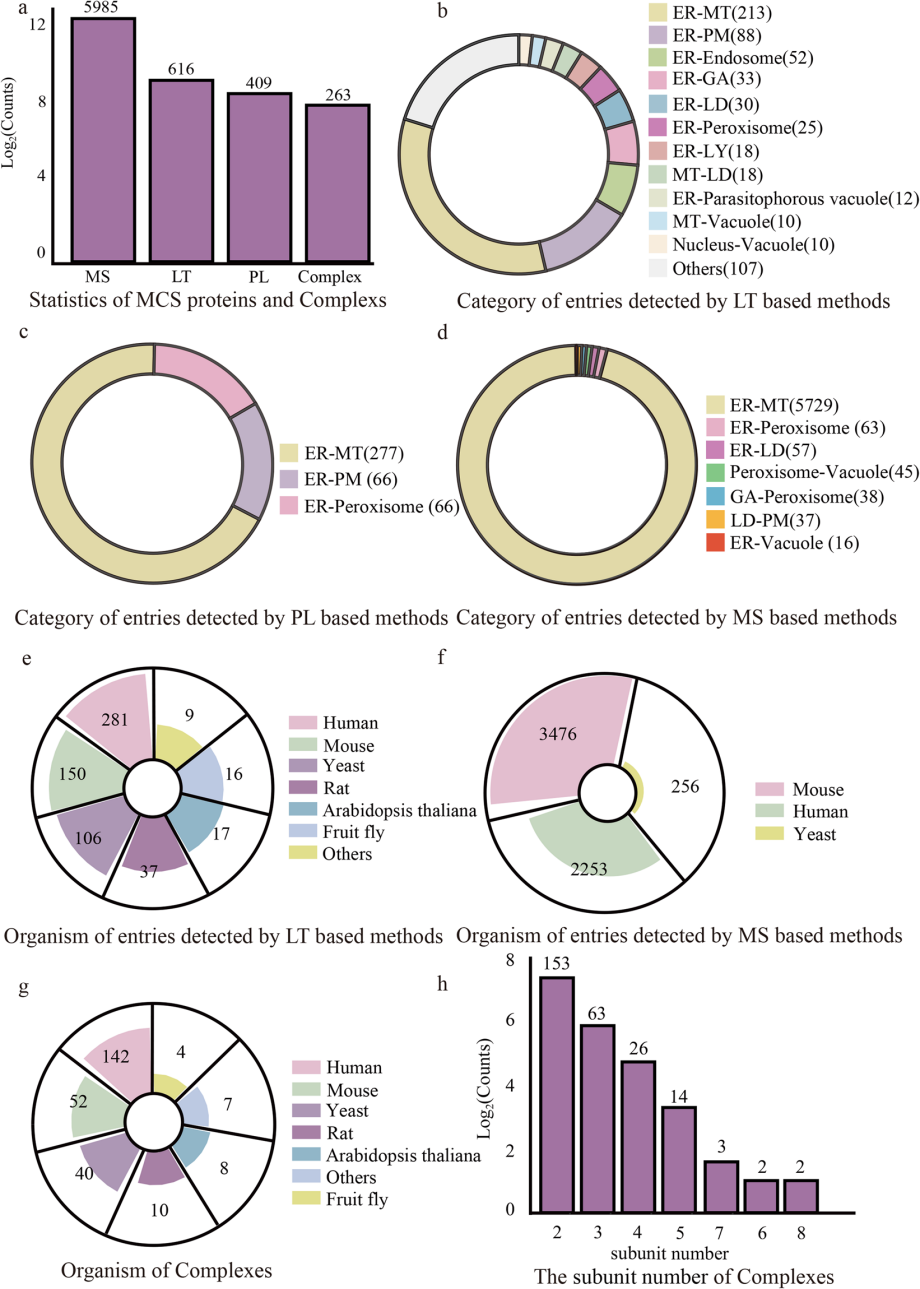
Data statistics of MCSdb. (**a**) Overview of MCS protein entries and complexes. (**b**) Category distributions of protein entries detected by LT-based methods. (**c**) Category distributions of protein entries detected by PL-based methods. (**d**) Category distributions of protein entries detected by MS-based methods. (**e**) Organismal distribution of protein entries detected by LT-based methods. (**f**) Organismal distribution of protein entries detected by MS-based methods. (**g**) Organismal distribution of complexes. (**h**) Subunit number distribution of complexes.

This current version of MCSdb documents 7010 manually curated MCS protein entries with experimental evidence (including 5985 entries detected by MS-based methods, 616 entries detected by LT-based methods and 409 entries detected by PL-based methods), referring to 24 organelles and 44 MCSs across 11 species; 263 complexes residing in MCSs are also included (Fig. 3a). The MCS category distributions of protein are shown in Fig. 3b–d. The protein entries detected by LT-based methods are distributed in multiple MCSs (Fig. 3b), most of which are located in ER-related MCSs (ER-MT: 213, ER-PM: 88 and ER-Endosome: 52, etc.). The protein entries detected by PL-based methods are divided into MCSs of ER-MT (277 proteins), ER-PM (66 proteins) and ER-Peroxisome (66 proteins). Over 95% protein entries detected by MS-based methods are located in the MCS of ER-MT (5729 proteins). All complexes, which were detected by LT-based methods, are distributed in multiple MCSs (data not shown). The organismal distribution of MCS proteins and complexes is shown in Fig. 3e–g. The protein entries detected by LT-based methods are distributed in 11 species, mostly human (281 proteins) and mouse (150 proteins) proteins (Fig. 3e). A total of 2253 human, 3476 mouse and 256 yeast protein entries were detected by MS-based methods (Fig. 3f). All the protein entries detected by PL-based methods are human proteins (data not shown). All the complexes are distributed in 11 species (Fig. 3g). The subunit number distribution of the complexes is shown in Fig. 3h, and most of the complexes are binary (153/263).

### Data submission

To acknowledge that the MCSdb collection may not include all proteins residing in MCSs, we offer a ‘Submit’ interface (https://cellknowledge.com.cn/mcsdb/submit.html) for researchers to submit new MCS proteins that have not yet been documented in the database. We will thoroughly review and update all submitted data in a timely manner.

## Technical Validation

The MCS protein entries in our database were carefully curated from peer-reviewed literature through manual selection, and we only included experimentally supported MCS proteins. In addition, all collected entries were evaluated and double-checked by at least two expert curators separately. Any discrepancies were resolved by consensus through discussion with the third expert curator.

To ensure the accuracy of our data, we collected detailed information on wet lab experiments used to identify MCS proteins, such as experimental methods and cell lines/tissues, from original articles. Additionally, we extracted original sentences from literature that explicitly described a protein’s role and residence in the MCS, providing further evidence for the accuracy of our data. All of these supporting data from the literature can be easily accessed on the website of our database: https://cellknowledge.com.cn/mcsdb/.

## Usage Notes

In addition to accessing the datasets via the Figshare repository^43^, MCSdb is also free available at https://cellknowledge.com.cn/mcsdb/. Moreover, the website provides a user-friendly ‘Help’ page that presents a step-by-step tutorial to assist users in manipulating, querying, and browsing the MCSdb database. On this ‘Help’ page, we not only offer guidance on maintaining data quality but also provide specific instances of errors as examples for users to reference (refer to the file of all revised entries.xlsx available on the ‘download’ page).

## Introduction to Revised Data

**Fig. 4:**
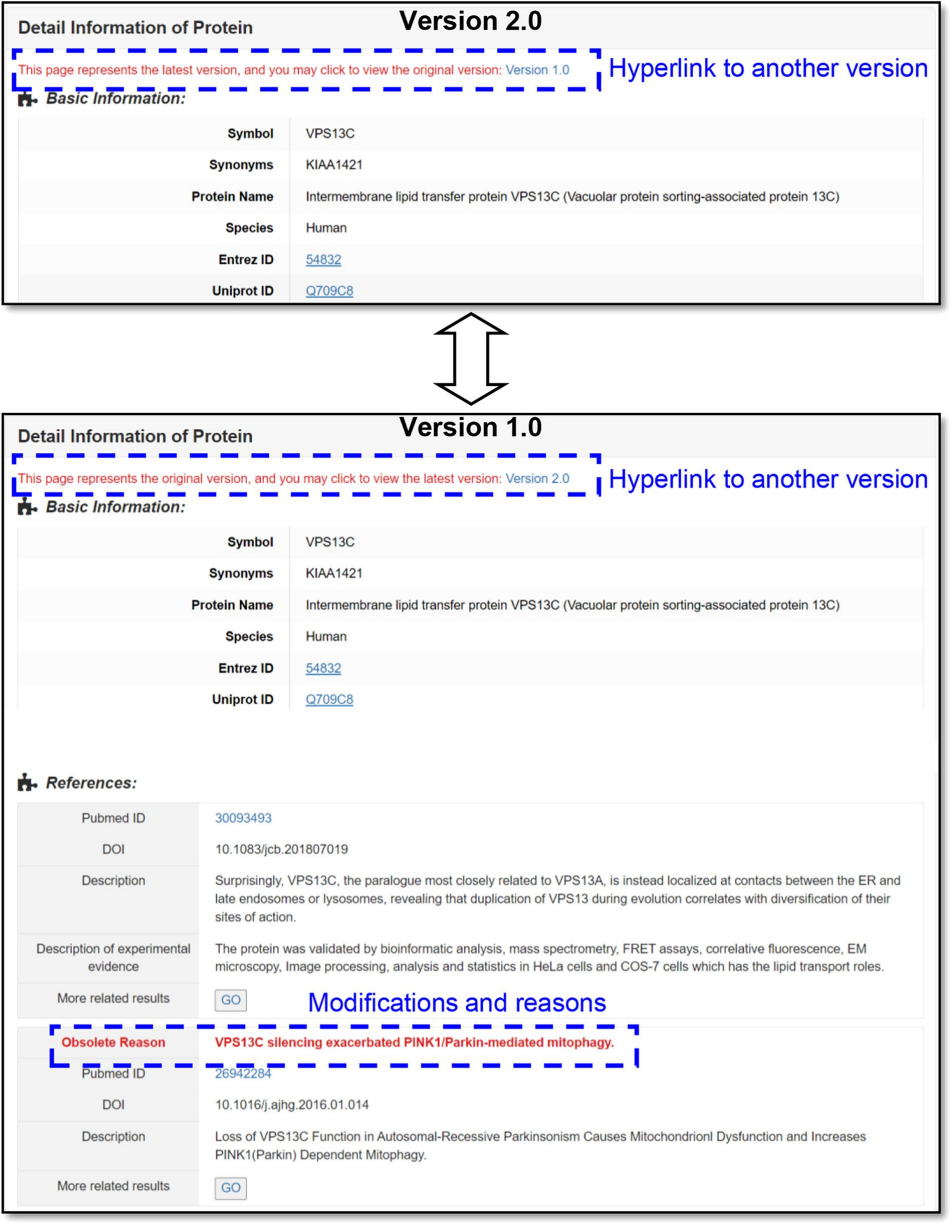
The differences between the two version pages of a modified entry.

During the data collection process, we continuously identified and corrected errors. To better safeguard users against encountering similar issues during literature searches and data collection, we have presented the errors found and their details during the revision process in the form of data tables on our database website (all revised entries.xlsx). In addition, to enhance user awareness and prevent similar mistakes, we have preserved all modified and obsolete entries in the database for user reference. Specifically, for modified entries, we offer two versions on the website: Version 1 and Version 2, with hyperlinks provided at the top of the detail pages for each version (Fig. 4). Version 1 is the original version, in which we have highlighted the specific modifications for user comparison and reference, while Version 2 presents the latest modified data. This approach allows users to easily see the changes made to the data. Moreover, we have separately displayed all obsolete entries in a list format on a dedicated “obsolete list” page, which includes three tables: the obsolete entries list, listing all obsolete protein entries; the table of obsolete Complex Entries list, listing all deleted complex entries; and the obsolete literatures list, listing all removed references.

## Data Availability

MCSdb is free available at Figshare^43^ and https://cellknowledge.com.cn/mcsdb/ to all users. The source code for the MCSdb website has been uploaded to GitHub: https://github.com/ZhangCellab/MCSdb.

## References

[1] Gottschling DE, Nyström T (2017). The Upsides and Downsides of Organelle Interconnectivity. Cell.

[2] Zhang Y (2021). CellCall: integrating paired ligand–receptor and transcription factor activities for cell–cell communication. Nucleic Acids Research.

[3] Huh JR, Veiga-Fernandes H (2020). Neuroimmune circuits in inter-organ communication. Nat Rev Immunol.

[4] Leitão AL, Costa MC, Gabriel AF, Enguita FJ (2020). Interspecies Communication in Holobionts by Non-Coding RNA Exchange. Int J Mol Sci.

[5] Zhang Y (2021). Cellinker: a platform of ligand-receptor interactions for intercellular communication analysis. Bioinformatics.

[6] Petkovic M, Oses-Prieto J, Burlingame A, Jan LY, Jan YN (2020). TMEM16K is an interorganelle regulator of endosomal sorting. Nat Commun.

[7] Elbaz Y, Schuldiner M (2011). Staying in touch: the molecular era of organelle contact sites. Trends Biochem Sci.

[8] Wu, H., Carvalho, P. & Voeltz, G. K. Here, there, and everywhere: The importance of ER membrane contact sites. **361**, eaan5835 (2018). 10.1126/science.aan5835 PMC6568312 30072511

[9] Scorrano L (2019). Coming together to define membrane contact sites. Nat Commun.

[10] Prinz WA, Toulmay A, Balla T (2020). The functional universe of membrane contact sites. Nat Rev Mol Cell Biol.

[11] Venditti R, Wilson C (2021). & De Matteis, M. A. Regulation and physiology of membrane contact sites. Current Opinion in Cell Biology.

[12] Chu Q (2019). Regulation of the ER stress response by a mitochondrial microprotein. Nat Commun.

[13] Ravindran, R. *et al*. Endosomal-associated RFFL facilitates mitochondrial clearance by enhancing PRKN/parkin recruitment to mitochondria. *Autophagy*, 1–14 (2022). 10.1080/15548627.2022.2052460 PMC9673925 35373701

[14] Petkovic M, O’Brien CE, Jan YN (2021). Interorganelle communication, aging, and neurodegeneration. Genes Dev.

[15] Herker E, Vieyres G, Beller M, Krahmer N, Bohnert M (2021). Lipid Droplet Contact Sites in Health and Disease. Trends Cell Biol.

[16] Lim CY (2019). ER-lysosome contacts enable cholesterol sensing by mTORC1 and drive aberrant growth signalling in Niemann-Pick type C. Nat Cell Biol.

[17] Hirabayashi Y (2017). ER-mitochondria tethering by PDZD8 regulates Ca(2+) dynamics in mammalian neurons. Science.

[18] Chao W, Quan ZOU (2021). A Machine Learning Method for Differentiating and Predicting Human-Infective Coronavirus Based on Physicochemical Features and Composition of the Spike Protein. Chinese Journal of Electronics.

[19] Wu Y (2017). Contacts between the endoplasmic reticulum and other membranes in neurons. Proc Natl Acad Sci USA.

[20] Lujan P, Angulo-Capel J, Chabanon M, Campelo F (2021). Interorganelle communication and membrane shaping in the early secretory pathway. Current Opinion in Cell Biology.

[21] Hoyer MJ (2018). A Novel Class of ER Membrane Proteins Regulates ER-Associated Endosome Fission. Cell.

[22] Jain A, Zoncu R (2022). Organelle transporters and inter-organelle communication as drivers of metabolic regulation and cellular homeostasis. Mol Metab.

[23] Sandhu J (2018). Aster Proteins Facilitate Nonvesicular Plasma Membrane to ER Cholesterol Transport in Mammalian Cells. Cell.

[24] Zhou Z (2020). Endoplasmic reticulum-associated degradation regulates mitochondrial dynamics in brown adipocytes. Science.

[25] Krawczyk HE (2022). SEED LIPID DROPLET PROTEIN1, SEED LIPID DROPLET PROTEIN2, and LIPID DROPLET PLASMA MEMBRANE ADAPTOR mediate lipid droplet-plasma membrane tethering. Plant Cell.

[26] Loewen CJ, Roy A, Levine TP (2003). A conserved ER targeting motif in three families of lipid binding proteins and in Opi1p binds VAP. Embo j.

[27] Loewen CJ, Levine TP (2005). A highly conserved binding site in vesicle-associated membrane protein-associated protein (VAP) for the FFAT motif of lipid-binding proteins. J Biol Chem.

[28] Loewen CJ, Young BP, Tavassoli S, Levine TP (2007). Inheritance of cortical ER in yeast is required for normal septin organization. J Cell Biol.

[29] Chao JT (2014). Polarization of the endoplasmic reticulum by ER-septin tethering. Cell.

[30] Murray R, Flora E, Bayne C, Derré I (2017). IncV, a FFAT motif-containing Chlamydia protein, tethers the endoplasmic reticulum to the pathogen-containing vacuole. Proc Natl Acad Sci USA.

[31] Wang X, Wen Y, Dong J, Cao C, Yuan S (2018). Systematic In-Depth Proteomic Analysis of Mitochondria-Associated Endoplasmic Reticulum Membranes in Mouse and Human Testes. Proteomics.

[32] Horner SM, Wilkins C, Badil S, Iskarpatyoti J, Gale M (2015). Proteomic analysis of mitochondrial-associated ER membranes (MAM) during RNA virus infection reveals dynamic changes in protein and organelle trafficking. PLoS One.

[33] Ma JH (2017). Comparative Proteomic Analysis of the Mitochondria-associated ER Membrane (MAM) in a Long-term Type 2 Diabetic Rodent Model. Sci Rep.

[34] Huang X, Jiang C, Yu L, Yang A (2020). Current and Emerging Approaches for Studying Inter-Organelle Membrane Contact Sites. Front Cell Dev Biol.

[35] Kwak C (2020). Contact-ID, a tool for profiling organelle contact sites, reveals regulatory proteins of mitochondrial-associated membrane formation. Proc Natl Acad Sci USA.

[36] Cho KF (2020). Split-TurboID enables contact-dependent proximity labeling in cells. Proc Natl Acad Sci USA.

[37] Go CD (2021). A proximity-dependent biotinylation map of a human cell. Nature.

[38] Jing J (2015). Proteomic mapping of ER-PM junctions identifies STIMATE as a regulator of Ca^2+^ influx. Nat Cell Biol.

[39] Hua R (2017). VAPs and ACBD5 tether peroxisomes to the ER for peroxisome maintenance and lipid homeostasis. J Cell Biol.

[40] Li S (2022). A new type of ERGIC-ERES membrane contact mediated by TMED9 and SEC. 12 is required for autophagosome biogenesis. Cell Res.

[41] Boutry M, Kim PK (2021). ORP1L mediated PI(4)P signaling at ER-lysosome-mitochondrion three-way contact contributes to mitochondrial division. Nat Commun.

[42] Wang C (2021). FUNDC1-dependent mitochondria-associated endoplasmic reticulum membranes are involved in angiogenesis and neoangiogenesis. Nat Commun.

[43] Pan X (2023). figshare.

[44] Licata L (2012). MINT, the molecular interaction database: 2012 update. Nucleic Acids Research.

[45] Calderone A, Castagnoli L, Cesareni G (2013). mentha: a resource for browsing integrated protein-interaction networks. Nat Methods.

[46] Li T (2017). A scored human protein-protein interaction network to catalyze genomic interpretation. Nat Methods.

[47] UniProt: the universal protein knowledgebase in 2021 (2021). Nucleic Acids Research.

[48] Sayers EW (2021). Database resources of the National Center for Biotechnology Information. Nucleic Acids Research.

[49] Kim S (2021). PubChem in 2021: new data content and improved web interfaces. Nucleic Acids Research.

[50] Uhlén M (2015). Proteomics. Tissue-based map of the human proteome. Science.

[51] Palasca O, Santos A, Stolte C, Gorodkin J, Jensen LJ (2018). TISSUES 2.0: an integrative web resource on mammalian tissue expression. Database.

[52] Huerta-Cepas J (2019). eggNOG 5.0: a hierarchical, functionally and phylogenetically annotated orthology resource based on 5090 organisms and 2502 viruses. Nucleic Acids Research.

[53] Penel S (2009). Databases of homologous gene families for comparative genomics. BMC Bioinformatics.

[54] Zdobnov EM (2020). OrthoDB in 2020: evolutionary and functional annotations of orthologs. Nucleic Acids Research.

[55] Schreiber F, Patricio M, Muffato M, Pignatelli M, Bateman A (2014). TreeFam v9: a new website, more species and orthology-on-the-fly. Nucleic Acids Research.

[56] Howe KL (2020). Ensembl Genomes 2020-enabling non-vertebrate genomic research. Nucleic Acids Research.

[57] Oughtred R (2021). The BioGRID database: A comprehensive biomedical resource of curated protein, genetic, and chemical interactions. Protein Sci.

[58] Liao PC (2022). Touch and Go: Membrane Contact Sites Between Lipid Droplets and Other Organelles. Front Cell Dev Biol.

[59] Li C (2021). Endoplasmic Reticulum-Plasma Membrane Contact Sites: Regulators, Mechanisms, and Physiological Functions. Front Cell Dev Biol.

